# Molecular markers of type II alveolar epithelial cells in acute lung injury by bioinformatics analysis

**DOI:** 10.1038/s41598-023-45129-9

**Published:** 2023-10-18

**Authors:** Xiaoting Yang, Jing Wang, Wei Liu

**Affiliations:** Emergency Department, The First Hospital of China Medical University, No.155 of North Street Nanjing, Heping District, Shenyang City, 110001 Liaoning Province China

**Keywords:** Gene expression, Genetic linkage study, Genetic markers, Genomics, Sequencing

## Abstract

In this study, we aimed to identify molecular markers associated with type II alveolar epithelial cell injury in acute lung injury (ALI) models using bioinformatics methods. The objective was to provide new insights for the diagnosis and treatment of ALI/ARDS. We downloaded RNA SEQ datasets (GSE109913, GSE179418, and GSE119123) from the Gene Expression Omnibus (GEO) and used R language package to screen differentially expressed genes (DEGs). DEGs were annotated using Gene Ontology (GO), and their pathways were analyzed using Kyoto Encyclopedia of Genes and Genomes (KEGG). DEGs were imported into the STRING database and analyzed using Cytoscape software to determine the protein network of DEGs and calculate the top 10 nodes for the hub genes. Finally, potential therapeutic drugs for the hub genes were predicted using the DGIdb database. We identified 78 DEGs, including 70 up-regulated genes and 8 down-regulated genes. GO analysis revealed that the DEGs were mainly involved in biological processes such as granulocyte migration, response to bacterial-derived molecules, and cytokine-mediated signaling pathways. Additionally, they had cytokine activity, chemokine activity, and receptor ligand activity, and functioned in related receptor binding, CXCR chemokine receptor binding, G protein-coupled receptor binding, and other molecular functions. KEGG analysis indicated that the DEGs were mainly involved in TNF signaling pathway, IL-17 signaling pathway, NF-κB signal pathway, chemokine signal pathway, cytokine-cytokine receptor interaction signal pathway, and others. We identified eight hub genes, including IRF7, IFIT1, IFIT3, PSMB8, PSMB9, BST2, OASL2, and ZBP1, which were all up-regulated genes. We identified several hub genes of type II alveolar epithelial cells in ALI mouse models using bioinformatics analysis. These results provide new targets for understanding and treating of ALI.

## Introduction

Acute lung injury (ALI) and acute respiratory distress syndrome (ARDS) are common causes of respiratory failure in critically ill patients, and their pathogenesis is not fully understood^1^. The pathological manifestations of ALI/ARDS are characterized by damage to pulmonary capillary endothelial cells and alveolar epithelial cells. This damage leads to an excessive production of inflammatory factors within lung tissue, resulting in respiratory distress, refractory hypoxemia, and non-cardiogenic pulmonary edema^2^. Regarding the pathogenesis of ALI/ARDS, the most widely discussed factors include the overactivation of the inflammatory response, increased permeability of both alveolar epithelium and vascular endothelium, as well as a decrease in the clearance of alveolar fluid in affected patients. However, further details and insights into this condition are still under investigation^3^. Despite progress in improving their diagnosis and treatment, the mortality rate of ALI/ARDS remains high, ranging from 30 to 40%^4^.

The alveolar epithelium, comprising both type II alveolar epithelial cells (ATII) and type I alveolar epithelial cells (ATI), governs fluid and ion transport, serving a pivotal function in preserving lung homeostasis. Additionally, these cells engage in fusion with the endothelial cells of capillaries, collectively forming a barrier crucial for lung ventilation^5^. However, various factors can induce damage to the epithelial cells during the early stage of ALI/ARDS, leading to disruption of the barrier function^6^. ATII cells play a pivotal role primarily in overseeing the proliferation and differentiation of ATI cells and in the recovery of lung epithelial function. However, they also possess the capacity to activate alveolar macrophages, which can exacerbate lung damage. Throughout this process, ATII cells can attract circulating immune cells, leading to the release of various mediators aimed at eliminating pathogens^7^. Consequently, the repair of damaged alveolar epithelial cells, especially the proliferation and differentiation of ATII cells, holds significant importance for the prognosis of ALI/ARDS^8^. Despite the identification of several biomarkers that can predict the severity of damage to alveolar epithelial cells and vascular endothelial cells in ARDS patients, these markers lack uniformity and specificity^9^. Hence, it is imperative to investigate related molecular markers specific to ATII cells in order to enhance our understanding of ARDS and explore novel therapeutic options.

Bioinformatics is an interdisciplinary field that combines computer science, information technology, novel mathematical algorithms, and statistical methods. It specializes in the analysis of biological experimental data, uncovering the hidden biological significance within the data. Moreover, it aims to develop novel data analysis tools for the acquisition and management of diverse information^10^. In comparison to other frequently used statistical methods, bioinformatics is known for its comprehensive and efficient approach. Recent advancements in gene sequencing at the mRNA level have opened up new avenues for investigating the mechanisms and treatment of diseases. The combination of chip technology and bioinformatics analysis further enables the exploration of diseases at the genetic level. While this method has been extensively employed for screening tumor targets at the genome level, there has been a limited number of bioinformatics studies focused on ALI/ARDS. Through these bioinformatics investigations, previous researchers have identified a multitude of genes associated with ALI/ARDS, with many of them being linked to inflammatory mechanisms^11,12^. Additionally, other studies have highlighted the significance of the body's immune response in relation to the prognosis of ALI/ARDS^13,14^. In this study, we employed bioinformatics analysis to explore the pivotal genes and molecular markers linked to ATII injury in various ALI models. We retrieved the necessary dataset from GEO, conducted data sorting and analysis using the R language, and identified the shared Differentially Expressed Genes (DEGs) in different ALI models. By subjecting these DEGs to Gene Ontology (GO) and Kyoto Encyclopedia of Genes and Genomes (KEGG) analyses, our aim was to uncover common patterns in gene expression. This approach enables us to potentially identify novel strategies for improving patient outcomes in the context of ARDS.

## Materials and methods

### Data sources

The Gene Expression Omnibus (GEO) is a gene expression database established by the National Center for Biotechnology Information (NCBI) in 2000. This database contains gene expression data submitted by research institutions worldwide, including gene chip and high-throughput sequencing data. We utilized GEO to retrieve the data needed for our study. We used “ATII”, “acute lung injury” and “ATII and acute lung injury”as key words to retrieve data sets. The screening criteria for selecting data were as follows: (1) model: acute lung injury mouse models, (2) data type: high-throughput sequencing and single-cell sequencing, (3) cell type: ATII cells, and (4) sample size: each dataset includes at least three samples in both the control and experimental groups. By setting these conditions, we aimed to obtain high-quality and relevant data for our study. The data set is screened again based on processing time.

### Research method

#### Identify differentially expressed genes (DEGs)

R language is a statistical programming language known for its capabilities in statistical computation, data mining, and data visualization. RStudio provides a supportive environment for executing code, creating visualizations, and more. In our study, we employed the R software to preprocess the data, which encompassed batch correction and standardization tasks accomplished through the use of the DESeq2 software package.To identify the differentially expressed genes (DEGs), we utilized the limma software package and removed overlapping genes from the datasets to obtain a final list of DEGs. The screening criteria for differential expression were set at an adjusted p-value < 0.05 and an absolute value of logarithmic fold change (|LogFC|) > 1^15^. These criteria ensured that only significant and biologically relevant genes were included in subsequent enrichment and protein network analyses.

#### Enrichment analysis of DEGs

To gain insights into the biological functions and pathways associated with the differentially expressed genes (DEGs), we performed Gene Ontology (GO) enrichment analysis (biological process, cellular component, and molecular function) and Kyoto Encyclopedia of Genes and Genomes (KEGG) pathway analysis using the clusterProfiler package in R software. We used the “ggplot2” package to generate graphical representations of the analysis results. The primary parameters were configured as follows: an enrichment significance threshold of (p-value) < 0.05 and a corrected p-value (q-value) < 0.05. Using these criteria, we were able to identify significantly enriched components within the analysis module.

#### Protein–protein interaction (PPI) network and identification of hub genes

To construct a protein–protein interaction (PPI) network for the differentially expressed genes (DEGs), we used the Search Tool for the Retrieval of Interacting Genes (STRING) database (http://string-db.org/). We uploaded the list of Differentially Expressed Genes (DEGs) into the STRING database, specifying the species as “Mouse,” and set the significance threshold at 0.4. Subsequently, the interaction network diagram of DEGs was automatically generated by the database. We then exported the node file for visualization using Cytoscape software^16^^.^

The CytoHubba plug-in and the MCODE plug-in are widely employed analytical tools within the software. In our analysis, we opted for the degree scoring algorithm in CytoHubba, as it is the most commonly used algorithm for identifying key genes. We set the criteria to select the top 10 key genes based on the degree ranking. Additionally, the MCODE plug-in was utilized to detect the most densely interacting module within the Protein–Protein Interaction (PPI) network. This was accomplished by calculating scores for each node. These identified core subnetworks, and the intersection between them, were identified as the hub genes^17^.

#### Identify drug candidates

DGIdb database is a valuable resource for predicting potential drugs for identified hub genes. By integrating drug-gene interaction data, it allows users to search for potential drugs that can target specific genes of interest^18^. The database provides information on FDA-approved drugs, experimental drugs, and investigational drugs, as well as their indications, target genes, and drug-gene interaction types^19^. Users can search for drugs based on gene symbols or drug names, and filter the results based on various criteria such as drug type, drug status, and interaction type. The database also provides links to other resources such as PubMed and ClinicalTrials.gov for further information on drugs and their clinical applications.

## Results

### Acquisition of data sets

According to the 2012 Berlin definition of ARDS, respiratory dysfunction must occur within one week of a known insult^20^. Therefore, we selected mouse models of ALI from datasets with time points within 72 h. Ultimately, we identified three gene chip datasets from the GEO database platform: GSE109913, which contains three samples of lung injury caused by lipopolysaccharide infection and control samples; GSE179418, which contains three samples of lung injury caused by Escherichia coli infection; and GSE119123, which contains five samples of lung injury caused by influenza virus. Each dataset includes an equal number of control samples. The detailed informationis illustrated in Table 1.

**Table 1:** Dataset base information.

	Model	Molding method	Processing time (h)	Sample	
				Treat	Control
GSE109913	Lipopolysaccharide	Endotracheal instillation	24	3	3
GSE179418	Escherichiacos	Endotracheal instillation	24	3	3
GSE119123	virus	Endotracheal instillation	72	5	5

### Differentially expressed genes of ATII in 3 datasets

**Figure 1:**
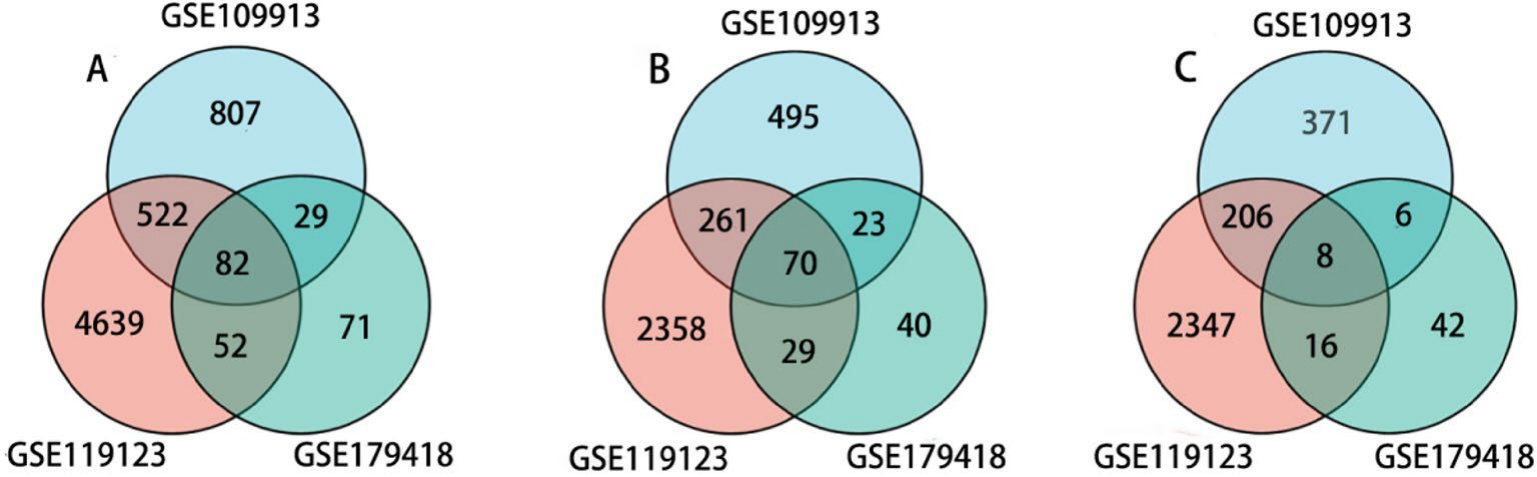
Expression of differential genes in GSE109913, GSE179418 and GSE119123. (**A**) All differentially expressed genes; (**B**) up-regulated genes; (**C**) down-regulated genes.

The R programming language package was utilized to analyze the aforementioned single cell RNA sequencing (RNA-seq) datasets. Using Venn diagrams, a total of 82 genes were identified, with 70 being up-regulated and 8 being down-regulated (Fig. 1A–C). Among them, four genes exhibited disparate expression patterns across different datasets and were thus excluded. Ultimately, 78 genes were identified as differentially expressed genes (DEGs) and were selected for further investigation.

### Go enrichment and KEGG pathway enrichment analysis of DEGs

**Figure 2:**
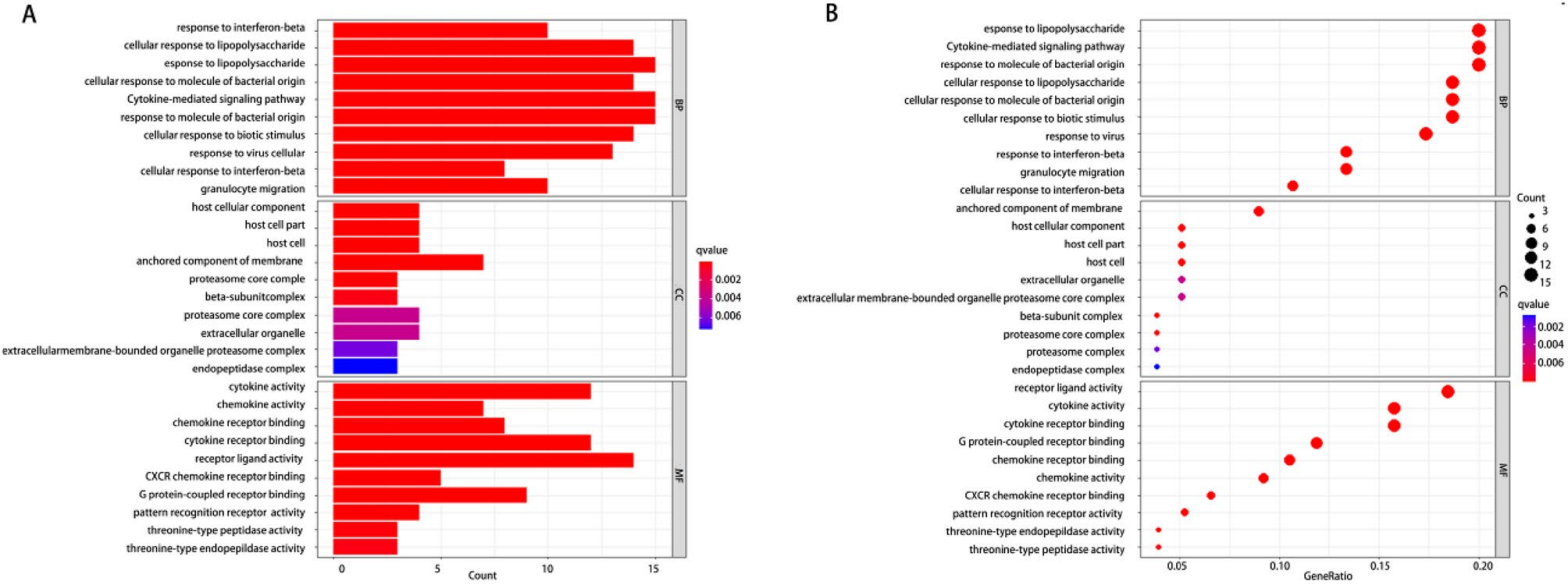
Gene ontology (GO) analysis (BP, CC, and MF) of DEGs. (**A**) Barplot, the abscissa is the number of enriched genes; (**B**) Bubble, the abscissa GeneRatio represents the proportion of enriched genes to the total number of genes.

**Figure 3:**
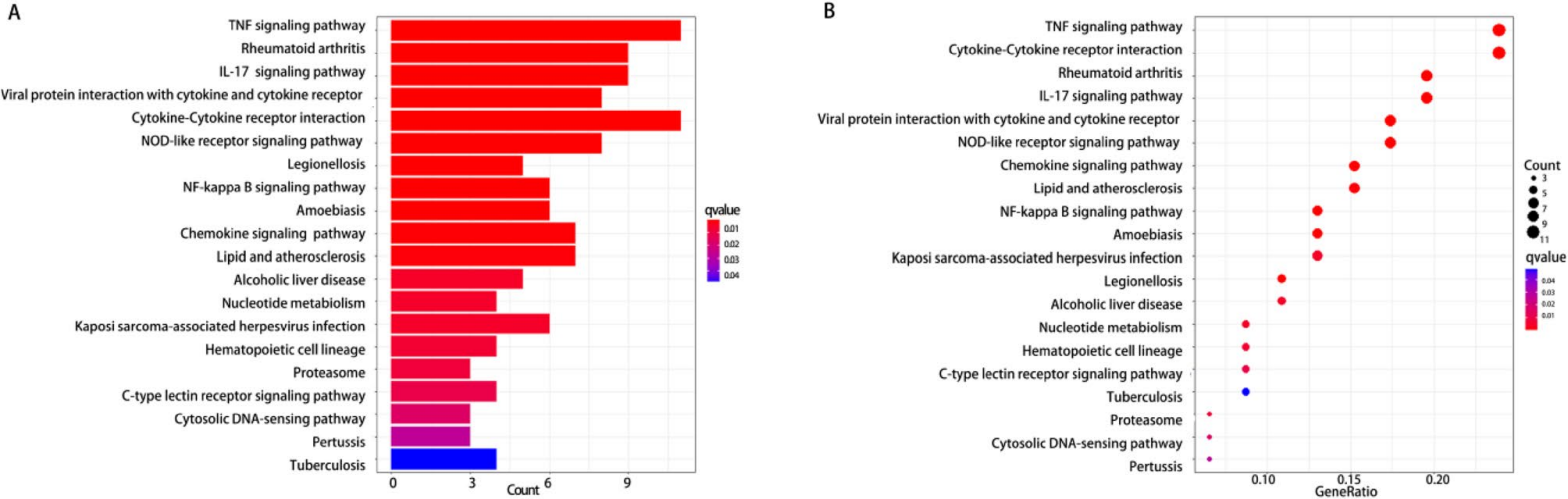
The KEGG enrichment analysis of DEGs. (**A**) Barplot, the abscissa is the number of enriched genes; (**B**) Bubble, the abscissa GeneRatio represents the proportion of enriched genes to the total number of genes.

The Go analysis was performed on the biological processes (BP), cellular components (CC), and molecular functions (MF) of the DEGs. The results revealed that the CC of these DEGs were primarily composed of host cell components, proteinome core complexes, and endopeptidase complexes, etc. The BP were significantly enriched in granulocyte migration, response to bacterial-derived molecules, cytokine-mediated signaling pathways, and others. These DEGs were also found to have cytokine activity, chemokine activity, and receptor ligand activity, and they can participate in related receptor binding, CXCR chemokine receptor binding, and G protein-coupled receptor binding, among others. Additionally, KEGG analysis indicated that the DEGs were mainly involved in TNF signaling pathway, IL-17 signaling pathway, NF-κB signaling pathway, chemokine signaling pathway, and cytokine receptor interaction signaling pathway (as shown in Figs. 2 and 3). The present study suggests that these pathways play a crucial role in the development of human ALI/ARDS. The NF-κB signaling pathway is intricately involved in various processes, including inflammatory responses, immune responses, apoptosis regulation, and stress responses. In the context of acute lung injury inflammation, NF-κB serves as a key transcription factor that, upon activation, promotes the expression of relevant inflammatory mediators. Additionally, it has the capacity to regulate the expression of genes associated with ALI^21^. Recent literature highlights the cytokine storm as a pivotal factor in inducing ARDS, with IL-17, TNF-α, and IL-6 being extensively discussed^22^. IL-17 plays a role in recruiting neutrophils, stimulating the release of various inflammatory cytokines, and amplifying the inflammatory response. Reducing the expression of TNF-α can benefit patients dealing with the disease. Chemokines also play a critical role in these processes, as they are produced by neutrophils and macrophages, and contribute to cell aggregation while maintaining local inflammation homeostasis. Considering these pathways, blocking the activity of relevant factors may be a viable approach for the treatment of ALI/ARDS.

### Protein–protein interaction (PPI) network and identification of hub genes

**Figure 4:**
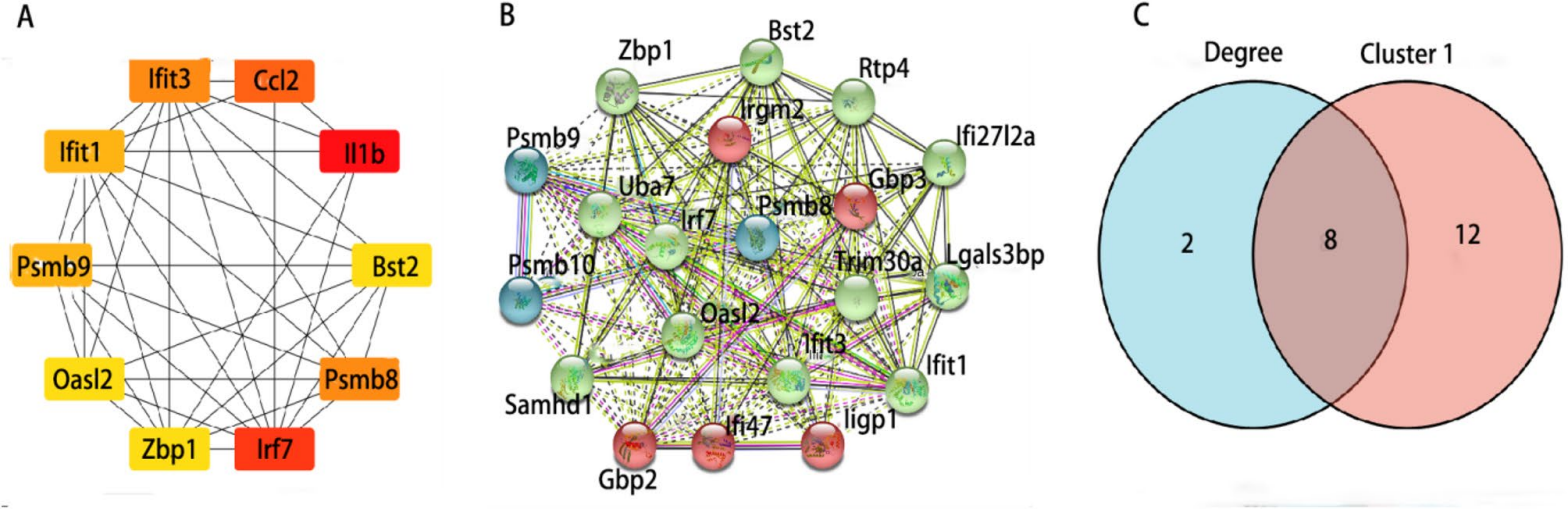
Protein–protein interaction (PPI). (**A**) Top 10 genes with the highest interaction degrees in PPInetwork analysis; (**B**) Cluster 1; (**C**) Hub genes.

Through the use of the STRING database and Cytoscape software, we identified the top 10 genes with the highest degree and the subnetwork with the highest MCODE score. The intersection of these results yielded eight hub genes: IFIT1, IFIT3, IRF7, PSMB8, PSMB9, BST2, OASL2, and ZBP1 (Table 2). These genes were identified as critical players in the PPI network and could potentially serve as therapeutic targets for ARDS. The visualization of this subnetwork is shown in Fig. 4.

**Table 2:** Hub genes.

(A) Expression of Hub genes in GSE109913			
Id	LogFC	P value	FDR
IFIT1	3.317081	2.1004e−15	9.7116e−14
IFIT3	3.331381	3.5990e−26	7.1959e−24
IRF7	3.575651	2.0854e−19	1.4693e−17
PSMB8	2.534904	5.6402e−14	2.1846e−12
PSMB9	1.936220	8.3409e−08	1.3502e−06
BST2	2.314502	4.1583e−14	1.6363e−12
OASL2	2.408518	2.4545e−07	3.6388e−06
ZBP1	4.438428	1.6038e−23	2.1973e−21

(B) Expression of Hub genes in GSE179418			
Id	LogFC	P value	FDR
IFIT1	1.120373	3.6228e−04	2.8116e−02
IFIT3	3.331381	9.9001e−05	1.4799e−02
IRF7	1.081377	9.9504e−05	1.4799e−02
PSMB8	1.340419	1.5478e−04	1.8019e−02
PSMB9	1.130255	8.3502e−05	1.3713e−02
BST2	1.635273	1.6969e−04	1.9038e−02
OASL2	1.480023	3.3362e−05	3.5552e−03
ZBP1	1.468907	3.5112e−05	9.7851e−03

(C) Expression of Hub genes in GSE119123			
Id	LogFC	P value	FDR
IFIT1	7.032258	6.2011e−62	2.1986e−58
IFIT3	6.415893	9.4179e−23	1.3465e−20
IRF7	7.348316	1.6653e−66	1.4761e−62
PSMB8	4.314184	5.1521e−28	1.1004e−25
PSMB9	3.822982	2.5269e−27	5.1490e−25
BST2	6.119503	1.0126e−45	7.8050e−43
OASL2	5.447088	9.7924e−46	7.8050e−43
ZBP1	7.150398	1.1486e−53	1.6969e−50

Note: logFC (fold change, the ratio of expression between the control group and the experimental group); P value (corrected p value); FDR (error rate, i.e. false positive).

### Identify drug candidates

Through the use of the DGIdb online database, we were able to identify drugs that are potentially relevant for the genes PSMB8 and PSMB9. However, we were unable to find any relevant drugs for the other hub genes. Table 3 displays the results of the drug prediction analysis for PSMB8 and PSMB9.

**Table 3:** Drug candidates combined with hub genes.

Drug names	Genes
Bortezomib	PSMB8, PSMB9
Carfilzomib	PSMB8, PSMB9
Ixazomib Citrate	PSMB8, PSMB9
Marizomib	PSMB8, PSMB9
Oprozomib	PSMB8, PSMB9

## Discussion and conclusions

In recent years, significant progress has been made in the research of ALI/ARDS, including epidemiology, pathogenesis, and pathophysiology. Studies on optimizing mechanical ventilation modes and fluid management have also brought benefits to clinical treatment. However, specific and effective therapeutic drugs for ALI/ARDS have not yet been identified^23^. With the rapid development of modern biotechnology, bioinformatics has gained more attention as researchers explore therapeutic options for ALI/ARDS at the molecular and cellular levels^24^. Numerous studies have shown that multiple pathogenic factors induce gene alterations during ALI/ARDS^25^. In this study, 78 co-expressed DEGs were screened from three ATII single-cell RNA sequencing datasets of ALI mouse models induced by three different pathogens. Finally, eight hub genes, including IRF7, IFIT1, IFIT3, PSMB8, PSMB9, BST2, OASL2, and ZBP1, were identified using bioinformatics methods. These hub genes are closely related to ATII injury during ALI, as indicated by protein network analysis.

A literature review shows that IRF7, IFIT1, IFIT3, BST2, and OASL2 are related to the immune response of the body. During lung injury, these genes are induced by interferon and participate in the IRF3–IFNAR–STAT1–IFIT1 signal pathway of pulmonary epithelial cells. They work together to regulate the activation of inflammatory cells and induce the death of infected cells^26^. The expression of IRF7 is increased by viral infection, tumor necrosis factor-α (TNF-α), and the inflammatory cytokine IL-1β. IRF7 also induces plasmacytoid dendritic cells and monocytes to produce the inflammatory cytokine IL-6, which participates in the occurrence and development of ALI/ARDS^27^. Proteomic studies of bronchoalveolar lavage fluid and ATII cells in ALI mouse models confirmed the important role of IRF7^26^. Excessive activation of IRF7 promotes the development of ALI/ARDS caused by Influenza A Avirus (IAV), and reducing IRF7 activity at local infection sites may be a new method to treat ALI/ARDS in IAV^28^. Studies have shown that IFN-induced protein with tetrapeptide repeats 3 (IFIT3) protects against lung injury caused by viral infection^29,30^. IFIT1 and IFIT3 induced by interferon can be used as relevant marker proteins of M1 polarization of pulmonary macrophages, and a useful marker of potential inflammatory diseases^31^. Bone marrow stromal cell antigen 2 (BST2) activates the NF-κB signal pathway, promoting the production of proinflammatory factors such as TNF-α, IL-1β, and IL-6^32^. As a member of the 2′-5′ oligoadenylate synthetase (OAS) family, OASL2 encodes an important antiviral protein and promotes the cleavage of viruses or infected cells^33^. At present, the role of OASL2 in ALI/ARDS has not been reported. Our results show that the expression of OASL2 is increased in different ALI models, suggesting that OASL2 may be a potential target worth further exploration in ALI/ARDS.

Proteasome subunit beta type-8 (PSMB8) and Proteasome subunit beta type-9 (PSMB9) are mainly expressed in monocytes and T lymphocytes, encoding proteasome β subunit. They are responsible for the degradation of proteasome after ubiquitination, promoting abnormal proliferation and anti-apoptosis of cancer cells^34^. The expression of PSMB8 and PSMB9 is significantly down-regulated in tumors^35^, but in inflammatory diseases, PSMB8 is highly expressed. By recognizing and degrading pathway repressor proteins in the NF-κB pathway, PSMB8 can promote the release of inflammatory mediators^36^. Selective inhibitors of PSMB8 can block and reduce the inflammatory reaction^37^. In the study of IAV-induced lung epithelial cell injury, researchers found that PSMB8 gene was up-regulated and inhibition of PSMB8 reduced the replication of influenza virus and attenuated lung epithelial injury^38^. Similarly, when analyzing the lung tissue and single-cell transcriptome results of patients with COVID19 infection, the results also showed that the expression of PSMB8 and PSMB9 was increased and related to the polarization of pulmonary macrophages^39^. Our results, together with others, indicate that PSMB8 and PSMB9 may play an important role in ALI/ARDS, providing a new target for treatment.

Z-DNA binding protein 1 (ZBP1), mostly expressed in CD8 + T lymphocytes, plays an important role in immune defense^40^. Previous reports revealed that ZBP1 could regulate the activation of the Nod-like receptor with pyrin domain-containing 3 inflammasome (NLRP3) through the RIPK3-caspase-8 axis, and promote the secretion of IL-1β and interleukin-18 (IL-18). Moreover, it could stimulate the apoptosis of necrotic cells at the infection site through pyroptosis^41^. Additionally, in an IAV-induced lung injury model of mice, ZBP1 could activate the NF-κB signaling and promote pro-inflammatory cytokines, resulting in the formation of neutrophil extracellular traps^42^. Our results showed that ZBP1 was highly expressed in three different ALI models, suggesting a prominent role in ALI/ARDS. However, the particular role and mechanism of ZBP1 still need further study.

Analyzing the final hub genes, we found that they all play an important role in regulating the immune system, which provides new ideas for the treatment of ALI/ARDS. Through online drug prediction, we obtained drugs primarily targeting genes PSMB8 and PSMB9, which are proteasome inhibitors mainly used in tumors and immune-related diseases^43^. An experiment demonstrated that bortezomib could improve lung function in an acute pancreatitis model of mice and reduce other complications^44^. Studies of other drugs are mainly used for tumor diseases such as hematological malignancies^45^, glioblastoma^46^, etc. Currently, there are no relevant reports on ALI/ARDS, so further research is needed in this field.

In summary, using bioinformatics methods, we screened and analyzed the common characteristics of differentially expressed genes of ATII in ALI/ARDS caused by different pathogens. The results of this study provide a basis for further exploring the pathogenesis, prognosis evaluation, and new drug targets of ALI/ARDS. The predicted related drugs need to be further investigated in animal experiments and clinical studies.

## Data Availability

The authors confirm that the data supporting the findings of this study are available within the article.
